# Effect of nondipper hypertension on coronary artery disease progression in patients with chronic coronary syndrome

**DOI:** 10.3906/sag-2011-225

**Published:** 2021-06-28

**Authors:** Deniz ELÇİK, Mustafa DURAN, Şaban KELEŞOĞLU, Zeki ÇETİNKAYA, Sibel BOYLUĞ, Rıdvan YURT, Ali DOĞAN, Mehmet Tuğrul İNANÇ, Nihat KALAY

**Affiliations:** 1 Department of Cardiology, Faculty of Medicine, Erciyes University, Kayseri Turkey; 2 Department of Cardiology, Ankara Research and Education Hospital, Ankara Turkey

**Keywords:** Coronary artery disease, nondipper hypertension, progression

## Abstract

**Background/aim:**

It has been suggested that there is a significant progress in coronary artery disease (CAD) by many pathophysiological mechanisms. Nondipper hypertension (NDH) has been shown to have higher target organ damage and have a higher rate of cardiovascular mortality and morbidity. In this study, we investigated the effect of nondipper hypertension on the progression of coronary atherosclerosis.

**Materials and methods:**

A total of 186 patients who underwent coronary angiography twice between 6 months and 3 years were included in the study. Coronary angiography was repeated on the admission day due to angina or positive exercise test and the patients were divided into groups.

**Results:**

Progression of coronary artery disease was detected in 58 of 186 patients. Seventy-one of the total patients were found to be nondipper hypertensive. Nondipper hypertension, hypertension, diabetes mellitus, low-density lipoprotein, and total cholesterol were found to be effective in the progression of CAD. Among these parameters, it was seen that nondipper hypertension and hyperlipidemia were the most important independent risk factors.

**Conclusion:**

Coronary artery disease is a progressive disease, and this progression depends on many reasons. In our study, we showed that nondipper hypertension is a new parameter that is effective in CAD progression.

## 1. Introduction

Although coronary artery disease (CAD) occurs in the early first stages of life, the onset and severity of the atherosclerotic process cannot be predicted. It is not known whether this process is associated to morphological progress [1]. The progression of atherosclerosis occurs by two mechanisms. The first is localized inflammation and thrombosis-related plaque rupture, with the artery filling partially or completely, and the other is progressive lipid accumulation that causes stenosis in the coronary artery [1,2]. Progress due to gradual lipid accumulation is characterized by the fact that the existing stenosis becomes more severe [1, 2]. Both types may occur at different rates in the same or different coronary arteries of any individual. Although there are many causes of CAD progressions, some causes and results are still not fully explained.

Cardiovascular parameters such as blood pressure, heart rate, coronary tone change with circadian rhythm throughout the day [3]. This circadian rhythm in blood pressure led to the creation of a new classification. In this classification made with ambulatory blood pressure monitoring, 10% or more decrease in blood pressure value measured at night compared to daytime value is defined as dipper hypertension, and less than 10% decrease as nondipper hypertension [4]. Patients with nondipper hypertension have been shown to have higher target organ damage and have a higher rate of cardiovascular mortality and morbidity [5,6]. Blood pressure variability (BPV) has been associated with measures of arterial stiffness and endothelial dysfunction, but whether BPV is directly associated with coronary atheroma progression-regression remains less well explored. However, its effect on coronary artery disease progression is unknown.

Lifestyle factors and the presence of existing diseases have been associated with the incidence of CAD. The importance of these factors in the progression of angiographic CAD has not been well studied. Our study aims to compare serial coronary angiograms of patients and to associate nondipper hypertension with CAD progression.

## 2. Materials and methods

### 2.1. Study population

Patients who underwent angiography twice due to angina or positive exercise test between April 2020 and October 2020 were included in the study. Local ethics committee approval was obtained for the study (2020/219). For the patients included in the study to be included in the study, the condition that the second angiography should be at least 6 months and a maximum of 3 years from the first angiography was sought. A new lesion in the second coronary angiography or 20% increase or ≥0.4 mm in MLD at the follow-up angiogram on QCA was accepted as possible coronary artery disease progression [7]. The groups were divided into two according to whether there was progression or not. Patients under 18 and over 75 years of age, those with kidney failure (need dialysis or those with glomerular filtration rate below 50 mL/min/1.73 m2), liver failure (chronic hepatitis, or aspartate aminotransferase-alanine aminotransferase basal value more than three times), a history of malignancy, and those who underwent coronary artery bypass surgery during these two coronary angiographies were not included in the study. 

Hypertension was defined as repeated arterial blood pressure measurements exceeding 140/90 mm Hg or treatment with antihypertensive medications for a known diagnosis of hypertension. Diabetes mellitus was diagnosed by fasting blood glucose ≥ 126 mg/dL, blood glucose > 200 mg/dL at any time, or a history of diabetes mellitus, including those treated with diet, oral medications, or insulin. Hyperlipidemia was defined as a baseline cholesterol level > 200 mg/dL and/or a low-density lipoprotein cholesterol level > 130 mg/dL or previously diagnosed and treated hyperlipidemia. 

While patients with chronic coronary syndrome who did not develop stent restenosis were included in the study, patients with stent thrombosis or restenosis were not included in the study [8]. Patients who had previously undergone angiography (6 months to 3 years) and who needed angiography according to the evaluation of the positive effort test or ischemia detected in myocardial perfusion scintigraphy were taken to angiography procedure for the second time were included in the study.

### 2.2. Ambulatory blood pressure measurement

Blood pressure measurements in the clinic were performed using a sphygmomanometer according to the European Society of Hypertension. Ambulatory blood pressure measurement (ABPM) was performed using the Microlife WatchBP device in the 24 h after the patient was included based on inclusion criteria using a cuff proper for the patient’s arm diameter. BP measurements were performed every 30 min during the daytime (between 07:00 and 22:00) and every 60 min during the night (22:00–07:00). Night, daytime, and 24-h BP measurements obtained from the measurements made for 24 h were analyzed. The percentage of drop in BP at night was calculated using “Night BP decrease (%) = (Daytime BP – Night KB) × 100 / Night BP” formula. If the mean BP measured during the night is less than 10% lower than the mean daytime measurement, these individuals were considered to be “nondipper”, and if the difference is 10% or more, to be “dipper”. According to these reduction rates, patients were grouped as divided as nondipper and dipper hypertension.

### 2.3. Coronary angiography

Selective coronary angiography was performed on all patients using the standard Judkins technique from the femoral or radial approach, according to the operator’s request. Nitrate was given to all patients to minimize their effects on coronary vasoconstriction and coronary lumen diameter. Sublingual glyceryl trinitrate (0.5 mg) or isosorbide dinitrate (5 mg) was given to patients presenting with stable angina (2–10 min before contrast injection). Coronary angiography analyses were performed by specialist cardiologists. Patients were followed angiographically in all epicardial coronary arteries (including subbranches) one individually. The first angiographies and last angiographies of the patients were recorded individually and quantitatively assessed with the use of Coronary Angiography Analysis System (CAAS).

### 2.4. Quantitative assessments

Each patient’s coronary arteriogram pair was evaluated by two experienced cardiologists who did not know the patient information and the timing of coronary angiography. Coronary artery segments were selected according to defined localizations. For each segment, measurements were made in the end-diastolic frames. Coronary strictures were measured where their severity appeared maximum. Coronary diameters were measured using the CAAS (Pie Data Medical) developed by Reiber and validated by different authors [9–11]. The body of the Judkins coronary catheter was used for calibration to determine absolute measurements in millimeters. Since the CAAS is less reliable for coronary arteries smaller than 1 mm in diameter, the stenosis assessment of these vessels was performed visually. Since the measurements of the images showing the most severe stenosis were considered to be sufficient, orthogonal images were not measured and averaged. For this evaluation, Toshiba Infx brand angiography device was used. In this evaluation, 20% shot of the old lesion in two cardiologist measurements was accepted as progression.

### 2.5. Laboratory assessments

At the time of admittance, tripotassium-ethylenediaminetetraacetic acid (EDTA)-based complete blood count, and from the blood samples taken into Isotherm-Gel Clot Activator-based biochemistry tubes, biochemistry parameters (fasting blood glucose, renal function tests, liver functions tests, total lipid profile), sedimentation, and complete blood count were studied for all patients. To assess the inflammatory status of the participants, the C-reactive protein (CRP) level was measured using a BN2 nephelometer (Dade Behring, Schwalbach, Germany). 

### 2.6. Statistical analysis

Statistical analyses were performed using SPSS Statistical Package version 22.0 for Windows (SPSS Inc, Chicago, IL, USA). Histogram, q-q values were examined. The Kolmogorov–Smirnov test was performed to assess data normality. The Levene test was used to assess variance homogeneity. Differences between groups with and without CAD were compared. Pearson’s χ2 analysis was applied for categorical variables. The Mann–Whitney *U* tests and the independent samples t-test were applied for continuous variables. Nonnormally distributed variables were expressed as median (range) and normally distributed variables were as mean ± standard deviation (SD), as appropriate. A p-value of <0.05 was considered to be statistically significant. Moreover, we used logistic regression analyses to observe the association between coronary progressions and the impact of the primary variable and of other variables that probably acted as confounders (hypertension, hyperlipidemia, diabetes mellitus, low-density lipoprotein, C-reactive protein, and nondipper hypertensions). All factors with a significance of p < 0.05 were entered a stepwise multivariate logistic regression analysis.

## 3. Results

A total of 186 patients who met the study criteria were included in the study. Coronary artery disease progression was detected in 58 (31.2%) of 186 patients. Seventy-one of the total patients were found to be nondipper hypertensive. Although the majority of the study population (74.7%) was male, no significant difference was found between the two groups according to sex. In the study, the average age was found to be 63 ± 7 and there was no significant difference between the two groups (p = 0.057). There was no significant difference between patients with coronary noreflow and normal coronary flow groups in terms of hemodynamic parameters, heart rate, systolic, and diastolic blood pressure.

In the history evaluation of the patients, a significant difference was found between the two groups in hypertension, diabetes mellitus, and hyperlipidemia, which are effective in the progression of CAD (p = ​​0.025, p = 0.003, and p < 0.001, respectively). There was no statistical difference between the two groups in terms of smoking and body mass index (Table 1). 

**Table 1:** Baseline demographic and clinical characteristics of the study groups.

Variable	Coronary artery disease progression		p-value
	Yes(n = 58)	No(n = 128)	
Age (years)	65 (58-71)	58 (52-70)	0.057
Male %	45 (78.5)	94 (73.4)	0.141
Hypertension %	31 (53.4)	40 (31.1)	0.025
Diabetes mellitus %	30 (51.7)	32 (25)	0.003
Smoking %	28 (43.1)	54 (42.2)	0.906
Hyperlipidemia %	40 (69)	45 (35)	<0.001
Nondipper hypertension	34 (58.1)	37 (28.9)	<0.001
BMI (kg/m2)	24.4 ± 2.7	25.9 ± 2.3	0.463
GFR mL/min/1.73 m2	94.2 ± 20.5	93.1 ± 20.5	0.756
Plasma creatinine	0.8 ± 0.2	0.8 ± 0.1	0.110
Aspartate aminotransferase	37.5 ± 32.8	37 ± 29.8	0.920
Alanine aminotransferase	26.5 ± 18.7	29.3 ± 24.9	0.440
Total protein	7.1 ± 2.5	6.7 ± 2.2	0.420
Plasma Albumin (g/dL)	3.9 ± 1.5	3.8 ± 1.9	0.741
Total cholesterol (mg/dL)	201.4 ± 46.3	176.3 ± 39	<0.001
Triglyceride (mg/dL)	161.8 ± 78.9	151.2 ± 81.5	0.402
HDL cholesterol (mg/dL)	37.5 ± 10.2	35.9 ± 13	0.383
LDL cholesterol (mg/dL)	117 ± 37.1	110.1 ± 34.3	0.013
Sodium (mg/dL)	139 ± 7.3	138.2 ± 5.9	0.415
Potassium (mg/dL)	4.3 ± 0.46	4.6 ± 3.1	0.420
Calcium (mg/dL)	8.8 ± 0.6	8.8 ± 1.1	0.990
White blood cell (103/µL)	16.4 ± 4.7	14.1 ± 3.1	0.330
Hgb (g/dL)	14.1 ± 2	14.3 ± 1.8	0.550
Platelet (103/µL)	258.4 ± 80.5	251.2 ± 80.9	0.560
Pletacrit (%)	0.26 ± 0.07	0.26 ± 0.03	0.640
RDW-CV (%)	15.1 ± 9.36	15.28 ± 8.96	0.900
CRP (mg/dL)	10.7 ± 3.1	6.2 ± 2.5	0.001

**Figure 1:**
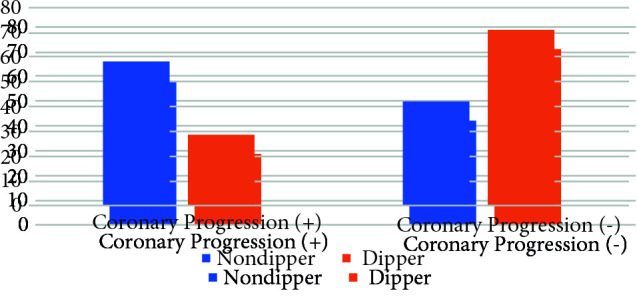
Nondipper hypertension rate in patients with CAD progression.

**Figure 2:**
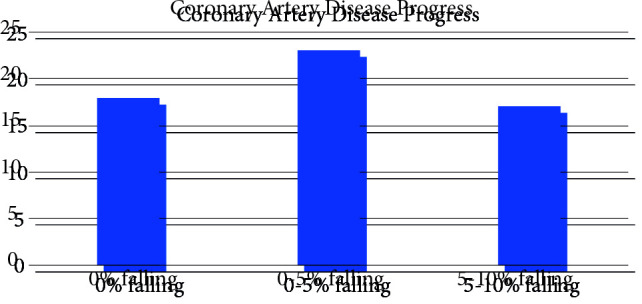
Patient evaluation of nocturnal blood pressure reduction rates in patients with CAD progression.

The main focus of this study is nondipper hypertension that is an independent risk factor in coronary artery progression regardless of the existing hypertension (Table 1). Nondipper hypertension was detected in 58.1% of patients with coronary progression and 28.9% of patients without progression (p < 0.001) (Figure 1). Another important finding of the study in terms of nondipper hypertension is the progression development independent of the nocturnal blood pressure reduction rate. Coronary progression developed in 18 patients with a 0% reduction in blood pressure at night, 23 patients with a drop between 0%–5%, and 17 patients with a decrease of 5%–10%. There was no statistically significant difference (Figure 2).

When the laboratory analyses were evaluated, some parameters in terms of biochemical parameters revealed that there was no significant difference between the groups such as creatinine, total protein, sodium, potassium, high-density lipoprotein (HDL-C), triglyceride levels, and glomerular filtration rate levels (p > 0.05) (Table 1). However, total cholesterol, low-density lipoprotein cholesterol (LDL-C), and CRP values were found to be significantly higher in the coronary artery progression group than in the nonprogressions group (Table 1). When the hematological parameters were examined between the two groups, no significant difference was found between the hemoglobin, platelet, and lymphocyte counts (p > 0.05).

It was determined that there was no statistically significant difference in all the above parameters examined between nondipper hypertensive patients and the dipper group except for hypertension, and hypertension was found to be statistically significant between the two groups (p: 0.020) (Table 2). 

**Table 2:** Baseline demographic and clinical characteristics of the dipper and nondipper groups.

Variable	Nondipper group		p-value
	Yes(n = 71)	No(n =115)	
Age (years)	63 (55-72)	59 (52-72)	0.067
Male %	56 (78.8)	83 (72.1)	0.386
Hypertension %	35 (49.2)	36 (31.3)	0.020
Diabetes mellitus %	28 (39.4)	34 (29.5)	0.201
Smoking %	27 (38.1)	55 (47.2)	0.106
Hyperlipidemia %	35 (49.1)	50 (43.4)	0.122
Plasma creatinine	0.8 ± 0.2	0.8 ± 0.1	0.112
Aspartate aminotransferase	41.5 ± 22.8	37 ± 26.8	0.224
Alanine aminotransferase	30.5 ± 25.7	27 ± 24.9	0.579
Total protein	7.1 ± 2.5	6.9 ± 2.3	0.242
Plasma albumin (g/dL)	3.8 ± 0.9	3.7 ± 1.1	0.685
Total cholesterol (mg/dL)	181.6 ± 32.6	184.3 ± 43	0.706
Triglyceride (mg/dL)	158.8 ± 83.4	147.5 ± 77.4	0.374
HDL cholesterol (mg/dL)	38.6 ± 9.2	39 ± 13.6	0.831
LDL cholesterol (mg/dL)	111 ± 27.1	114.1 ± 38.3	0.572
Sodium (mg/dL)	138 ± 7.3	138.2 ± 6.9	0.884
Potassium (mg/dL)	4.2 ± 0.4	4.6 ± 0.1	0.318
Calcium (mg/dL)	8.8 ± 0.6	8.8 ± 1.1	0.989
White blood cell (103/µL)	16.3 ± 4.7	14.1 ± 3.1	0.310
Hgb (g/dL)	14.1 ± 2	14.3 ± 1.8	0.345
Platelet (103/µL)	258.4 ± 80.5	251.2 ± 80.9	0.876
Pletacrit (%)	0.26 ± 0.07	0.26 ± 0.08	0.640
CRP (mg/dL)	9.7 ± 1.2	7.6 ± 1.9	0.076

In our study, we determined that several parameters are effective in CAD progression in addition to nondipper hypertension. Since the variables were found to affect the CAD progression between the groups, the effects of multiple variables on CAD progression were analyzed by univariate and multivariate logistic regression analyses. At univariate analysis, hyperlipidemia (odds ratio (OR) 4.620, 95% confidence interval (CI) 2.441–8.855; p = 0.001), hypertension (OR 2.200, 95% CI 1.181–4.100; p = 0.013), diabetes mellitus (OR 2.636, 95% CI 1.391–4.997; p = 0.003), CRP (OR 1.059, 95% CI 1.016–1.104; p = 0.007), low density lipoprotein (OR 1.011, 95% CI 1.002–1.020; p = 0.016), and nondipper hypertensions (OR 2.986, 95% CI 1.591–5.604; p = 0.001) were independent risk factors of progression in patients with CAD progressions (Table 3). Multivariate analysis showed that hyperlipidemia (OR 3.476, 95% CI 1.590–7.601; p = 0.002), diabetes mellitus (OR 2.522, 95% CI 1.115–5.700; p = 0.026), low density lipoprotein (OR 1.017, 95% CI 1.005–1.029; p = 0.006), CRP (OR 1.069, 95% CI 1.008–1.133; p = 0.0025), and nondipper hypertensions (OR 3.742, 95% CI 1.680–8.334; p = 0.001) were effective and that the age had some effect if not statistically significant. Based on these results, the two most effective parameters in the progression of CAD were hyperlipidemia and nondipper hypertension in the history. 

**Table 3:** Effects of multiple variables on the coronary artery progression in univariate and multivariate logistic regression analyses.

	Univariant				Multivariant	
Variables	Odds ratio	95% CI	p-value	Odds ratio	95% CI	p- value
Age	1.011	0.983–1.040	0.434			
BMI	0.937	0.867–1.014	0.104			
Male	0.759	0.373–1.543	0.446			
Hyperlipidemia	4.620	2.441–8.855	0.001	3.476	1.590–7.601	0.002
HT	2.200	1.181–4.100	0.013	1.572	0.735–3.525	0.272
DM	2.636	1.391–4.997	0.003	2.522	1.115–5.700	0.026
CRP	1.059	1.016–1.104	0.007	1.069	1.008–1.133	0.025
LDL	1.011	1.002–1.020	0.016	1.017	1.005–1.029	0.006
Nondipper HT	2.986	1.591–5.604	0.001	3.742	1.680–8.334	0.001
Creatinine	3.338	0.732–15.126	0.120			

## 4. Discussion

This is the first study conducted with nondipper hypertension in the development of CAD progression. In this study, it has been shown that nondipper hypertension is an independent risk factor for the progression of coronary artery disease. Moreover, it is effective in hyperlipidemia, diabetes mellitus, and LDL on the progression of CAD.

Atherosclerosis is a process characterized by intimal thickening in the arterial vasculature, accompanied by inflammatory cell infiltration, smooth muscle cell proliferation, excessive accumulation of oxidized low-density lipoproteins, and plaque development [12]. These parameters are known to play an important role in the formation and growth of atherosclerotic plaque, including high cholesterol, diabetes, and high blood pressure [13]. In coronary artery disease progression, smoking affects factors other than hypertension and diabetes. In the study conducted by Sahin et al., it was shown that the copper level was high in the blood in the group with CAD progression, and the chromium level was low compared to the group without progression [14].

The relationship of high pressure change with the measurements of arterial stiffness and endothelial dysfunction suggests a contribution to vascular function changes [15,16]. In addition, hypertension was shown as a risk factor in the development of coronary calcium progression [17]. In these studies, the effectiveness of variable high pressure on endothelial dysfunction was mentioned and it was said that it would lead to pathophysiology. In nondipper hypertension, it can be thought to act with this mechanism, as there is variable high pressure. In addition, while analyzing the relationship between antihypertensive therapy and cardiovascular risk, they also examined whether blood pressure was effectively reduced by antihypertensive therapy [17]. It was found that the risk of stroke and coronary events increased in participants with blood pressure falling to normal levels compared to participants who achieved normal blood pressure levels without treatment, but the rapid CAD progression was not accelerated. Of course, in this study, arterial blood pressure was investigated, not nondipper hypertension. In other words, arterial hypertension was shown to be a persistent risk in this study [18]. In our study, similar to this study, the presence of nondipper phenomena in patients with or without arterial hypertension increases coronary progression. Vascular endothelial damage is one of the first and most important steps in the coronary progression process. After endothelial damage, lipid accumulation starts to increase in the same area, and tissue proliferation increases. Endothelial growth factors are released by the cells that accumulate in this region and cause the proliferation of smooth muscle cells [19]. In our study, we believe that this is due to occasional attacks of hypertension and does not indicate the expected decrease. A strong link between atherogenic risk factors and endothelial dysfunction has been identified. By down-regulation of endothelial nitric oxide synthase (NOS) expression, oxidized LDL-C reduces receptor-mediated NO release, increases superoxide anion production, and NO inactivation causes a strong impairment of endothelial function. Disruption of NO production or activity predisposes individuals to many diseases targeting the cardiovascular system such as CAD, HT, heart failure, kidney failure, diabetes mellitus, metabolic syndrome, and obesity, and accelerates atherosclerosis [20]. Particularly in hypertensive patients, impairment of nitric oxide bioavailability causes impairment of endothelium-mediated vasodilatation, and this may be an indicator of atherosclerosis and progression in atherosclerosis [21]. 

“Nondipper” blood pressure is seen in approximately 25% of the hypertensive cases, and when subgroups such as diabetics are included, the prevalence increases even more [22]. It is known that there is a direct proportion between blood pressure level, and the grade of endothelial dysfunction, vascular damage, and end-organ damage [5]. Individuals with nondipper blood pressure have been found to have more frequent end-organ damage (ventricular hypertrophy, microalbuminuria, decreased arterial compliance, etc.), and cardiovascular morbidity and mortality [5,6]. Nondipper hypertension, as in our study, plays an important role in atherosclerosis because it is a branch of hypertension [5]. In their studies comparing nondipper and dipper patient groups for endothelial dysfunction, Higashi et al. evaluated the 24-h urinary excretion of nitric oxide end-product nitrite/nitrate and cyclic guanosine monophosphate as an indicator of endothelial dysfunction, and as a result, 24-h urinary nitrite/nitrate and cyclic guanosine monophosphate levels were found to be significantly lower in the nondipper patient group [23]. This data strengthens our hypothesis and should be regarded as evidence that nondipper hypertension causes endothelial dysfunction. We think that exposure of the endothelium to higher pressure for a longer period due to insufficient blood pressure drop at night may increase the progression of CAD more by causing more endothelial damage and vascular inflammation in the nondipper group.

In the studies performed by Smith et al. and Wild et al., the patients who underwent percutaneous coronary intervention had approximately 25%–30% DM [24,25]. In our study, this rate was similar to other studies, and 26.8% of patients with a history of percutaneous coronary intervention had DM. Those with diabetes mellitus have a higher rate of thrombotic events than those who do not and require a more frequent reintervention procedure [26]. In our study, there was a statistically significant difference in CAD progression compared to those without diabetes mellitus. It is important that we were unable to access data on medical treatments received before and after discharge from patients without diabetes mellitus and from those with diabetes mellitus. In addition, the duration of diabetes mellitus and whether they were under glycemic control could not be evaluated. As a result, these factors may be considered to increase or decrease CAD progression.

## 5. Conclusion

Coronary artery disease is a progressive disease and this progression depends on many reasons. In our study, we showed that it has an effect on nondipper hypertension as a new parameter that affects this progression. Our results show that treatment of nondipper hypertension may reduce the additional contribution to progress.

## 6. Limitations

The most important limitation of study is the number of patients and being a single-center study. In addition, the use of intracoronary ultrasound, which gives better results in CAD progression, will be more qualitative. 
